# Revolution of CAR Engineering For Next-Generation Immunotherapy In Solid Tumors

**DOI:** 10.3389/fimmu.2022.936496

**Published:** 2022-07-12

**Authors:** Tao Yu, Shao-kun Yu, Yan Xiang, Kai-Hua Lu, Ming Sun

**Affiliations:** ^1^ Department of Oncology, The First Affiliated Hospital of Nanjing Medical University, Nanjing, China; ^2^ Suzhou Cancer Center Core Laboratory, Suzhou Municipal Hospital, Gusu School, The Affiliated Suzhou Hospital of Nanjing Medical University, Suzhou, China

**Keywords:** CAR-T cells, solid tumor, receptors, co-stimulatory domains, cytokine, immune cells

## Abstract

Chimeric antigen receptor (CAR)-T cells have enormous potentials for clinical therapies. The CAR-T therapy has been approved for treating hematological malignancies. However, their application is limited in solid tumors owing to antigen loss and mutation, physical barriers, and an immunosuppressive tumor microenvironment. To overcome the challenges of CAR-T, increasing efforts are put into developing CAR-T to expand its applied ranges. Varied receptors are utilized for recognizing tumor-associated antigens and relieving immunosuppression. Emerging co-stimulatory signaling is employed for CAR-T activation. Furthermore, other immune cells such as NK cells and macrophages have manifested potential for delivering CAR. Hence, we collected and summarized the last advancements of CAR engineering from three aspects, namely, the ectodomains, endogenous domains, and immune cells, aiming to inspire the design of next-generation adoptive immunotherapy for treating solid tumors.

## Introduction

**Figure 1:**
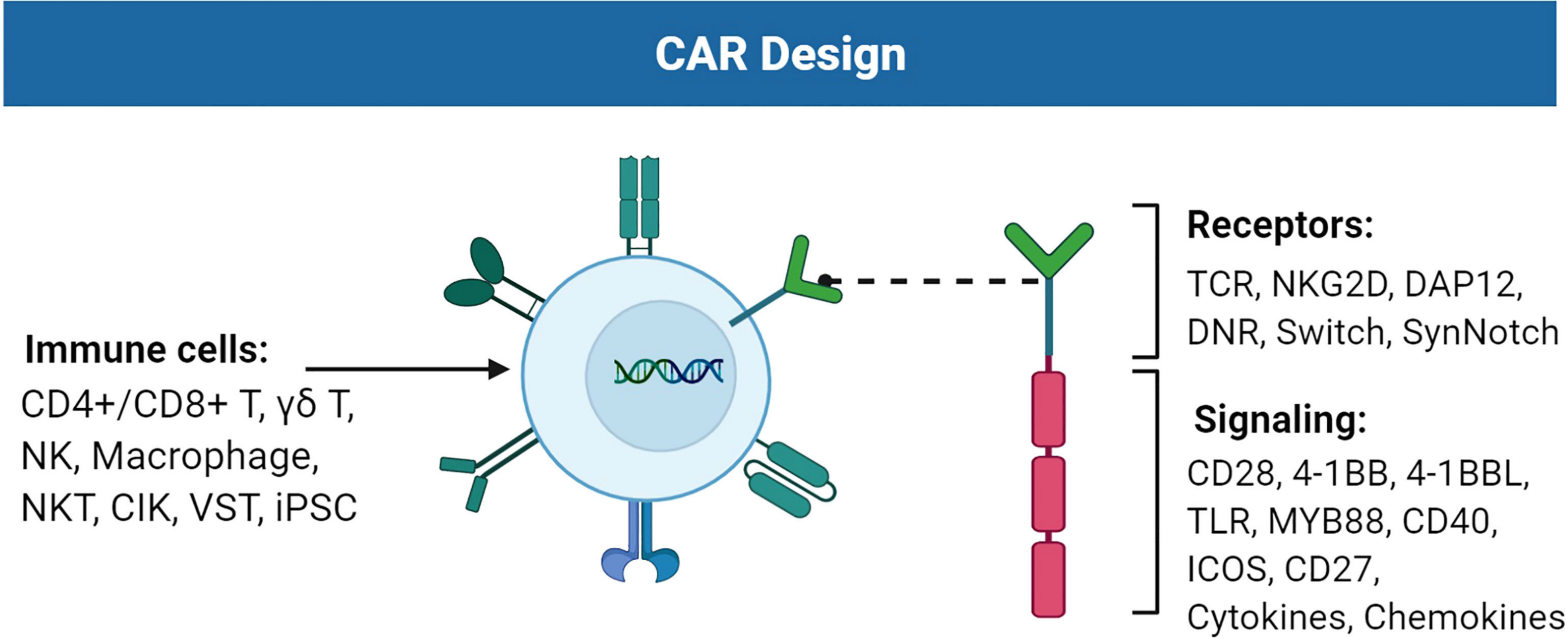
Improvements of the chimeric antigen receptor (CAR) design. CAR consists of receptors and signaling suitable for TAA recognition and cell activation. Immune cells such as T cells, NK cells, and macrophages can carry CAR to perform cytotoxic activities against tumor cells. Figure created using BioRender.com.

Over the past decades, the breakthrough of gene engineering and editing technologies has led to the fast improvement of chimeric antigen receptor (CAR)-T therapy. CAR-T cells combine the antitumor abilities of T cells with the recognition abilities of single-chain variable fragments (scFvs) (1). The extracellular scFv can recognize tumors expressing specific tumor-associated antigens (TAAs) in a major histocompatibility complex (MHC)-independent manner. Subsequently, the intracellular domain CD3ζ activates CAR-T to secrete cytokines and eradicate cancer cells regardless of their genomic mutations (2). CAR-T cells targeting CD19 have been approved for treating refractory B-cell malignancies. The designs of CARs have experienced four generations. The first-generation (1G) CAR only carries scFvs and immunoreceptor tyrosine-based activation motifs (ITAMs) of CD3ζ (3). The second- and third-generation (2G/3G) CARs are innovated by adding co-stimulatory molecules from the B7 family and tumor necrosis factor receptor (TNFR) subfamily, such as CD28, DAP10, CD134 (OX40), and CD137 (4-1BB) (4). Co-stimulatory fragments enable T cells to secrete cytokines and differentiate upon repeated contact with antigens (5, 6). CAR-T therapy has been widely utilized for hematological tumors; however, the bulk of difficulties constrain the application of CAR-T in solid tumors. Clinical trials of CAR-T targeting different TAAs in solid tumors have been thoroughly listed elsewhere. Here, we review and summarize the advancements of CAR engineering, T cells, or other immune cells that express CARs, with a particular focus on sophisticated engineering strategies of CAR design to improve the next-generation adoptive immune cell therapy for treating solid tumors (**Figure 1**).

## CAR-T challenges and improvements in solid tumors

The challenges of CAR-T therapy for solid tumors include trafficking, tumor recognition and killing, proliferation and persistence, immunosuppressive tumor microenvironment (TME), and control of CAR-T (7). One of the major difficulties is that heterogeneous solid tumors rarely express tumor-specific antigens, which prevents the recognition of CAR-T and induces on-target/off-tumor effects (8). Moreover, the conventional CAR structure has shortcomings. Sustained weak CD3ζ motif activation can raise CAR tonic signaling, leading to clustering of CAR molecules and causing T-cell exhaustion (9). Impaired T cells highly express inhibitory markers such as programmed cell death 1 (PD-1), TIM-3, and LAG-3 and are prone to apoptosis (10). Excessive activation of CAR-T following interleukin (IL)-6 overproduction may induce cytokine release syndrome (CRS), which is the most significant treatment-related toxicity (11). In addition, some patients lacking sufficient T cells require allo-CAR-T therapy (12). Endogenous T-cell receptors (TCRs) on allogeneic CAR-T may cause the graft-versus-host disease (GvHD), and immune rejection of T cells would reduce CAR-T efficacy. Although disruption of human leukocyte antigen (HLA) loci on CAR-T might avoid the rejection, it would reciprocally expose CAR-T to the rejection of natural killer (NK) cells.

To overcome the above problems, countermeasures based on optimal targets or novel CAR construct have been developed (13). Firstly, increasing research is conducted to identify recognizable and ideal TAAs for solid tumors, such as mesothelin, GD2, and MUC1 (14). Sequencing and proteomics have also been used for identifying targetable neoantigens (15). At the same time, the newly designed CAR can target multiple antigens with specific antibodies. For instance, camelids use a variable heavy chain domain (V_H_H), also known as nanobody (Nb), for combining antigens (16, 17). Nbs have the potential to serve as antigen recognition domains in CAR-T due to their small size, high affinity, optimal stability, and manufacturing feasibility (18). Nbs display a large convex paratope, which enables them to approach epitopes that are inaccessible for classical antibodies (19). Nbs are specifically suitable for bi-specific CARs containing two tandem antigen recognition domains (20). VH and VL in two independent scFvs may mispair and aggregate, while using Nbs can avoid domain swapping between variable and constant domains. Multiple antigens of solid tumors such as HER2, programmed cell death 1 ligand 1 (PD-L1), and EGFR could be chosen as targets of Nb-based CAR-T (21–24). Secondly, activation and co-stimulation domains can be optimized. For example, CD19 CAR-T with mutated first and third CD3ζ ITAMs was resistant to apoptosis and presented better antilymphoma efficacy than that with normal ITAMs (25, 26). In addition, improving co-stimulatory molecules seems promising. Merits and demerits of CD28 and 4-1BB have been deeply discussed (27). Substituting CD28 with 4-1BB can avert the CAR tonic signaling (28). A broad range of molecules, such as OX40, CD27, and inducible T-cell co-stimulator (ICOS), are also possible co-stimulators (29). Furthermore, distinctive immune cells beyond T cells delivering CAR might achieve unexpected success (30–32). Interestingly, recent studies have used other immune-activating receptors instead of CD3ζ for CAR design and optimization and have gotten surprising results. Hence, the structural characteristics of CAR-T significantly impact the functionality of adoptive immunotherapy in solid tumors (8, 13). Specifically, improvements in extracellular receptors, intracellular signaling, and carrier immune cells are summarized to enlighten investigators.

## Developments of extracellular receptors

**Figure 2:**
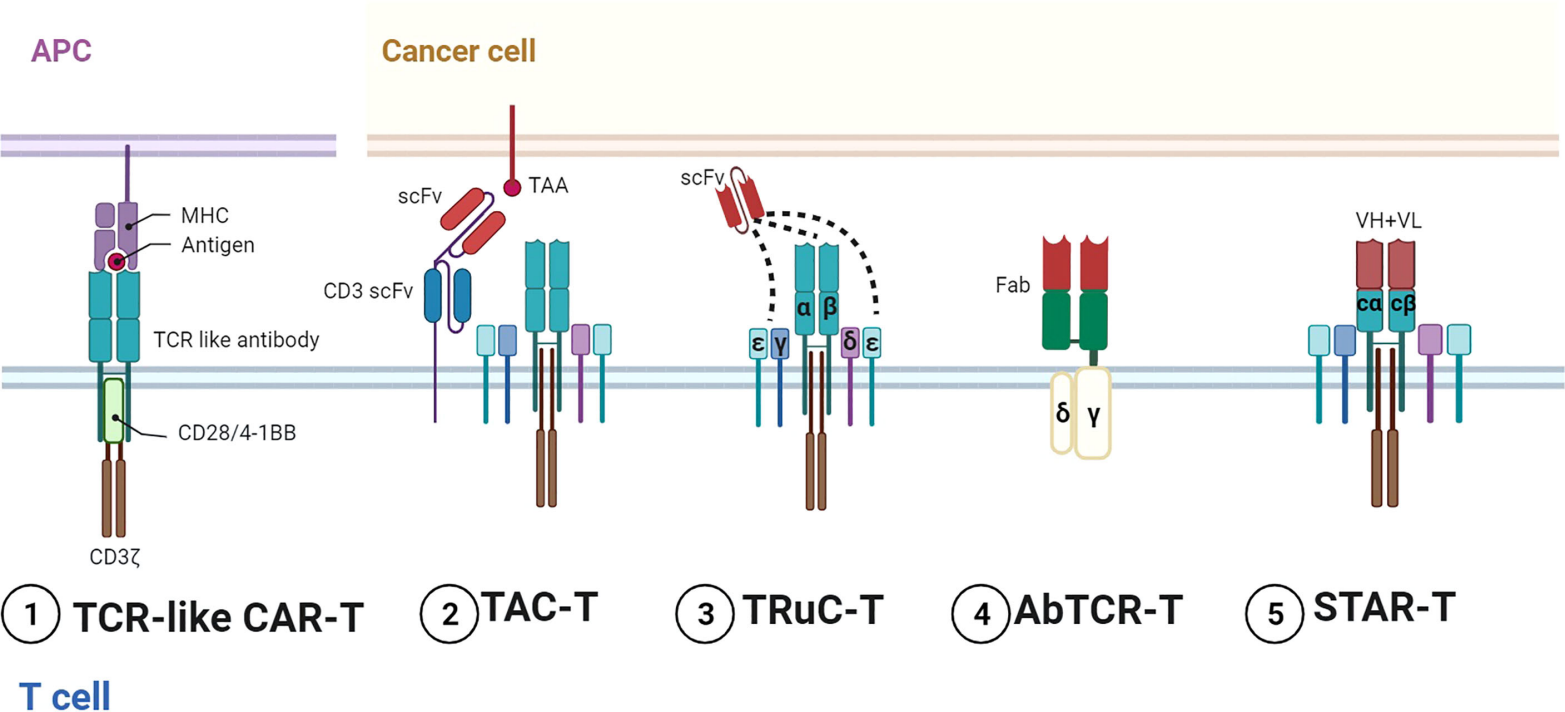
Representative designs of TCR–CARs. Diagrams of TCR–CARs with different configurations for tumor antigen recognition and cell activation. 1) TCR-like CAR-T utilized TCR-like antibodies for recognition and conventional stimulatory domains for activation. 2) TAC-T combined TCR with specific TAAs by two scFvs. 3) TRuC T bound scFvs to different parts of TCR and CD3. 4) AbTCR-T incorporated Fabs and γδ TCR. 5) STAR-T contained VH and VL with TCR-Cα/β. Figure created using BioRender.com.

Many studies have reported the developments of scFvs (33). Here, we mainly focused on distinctive receptors in extracellular domains (**Figures 2** **–** **4**). They could recognize multiple targets, induce downstream activation, and even reverse inhibitory signaling into the activation pathway (**Tables 1**, **2**).

**Figure 3:**
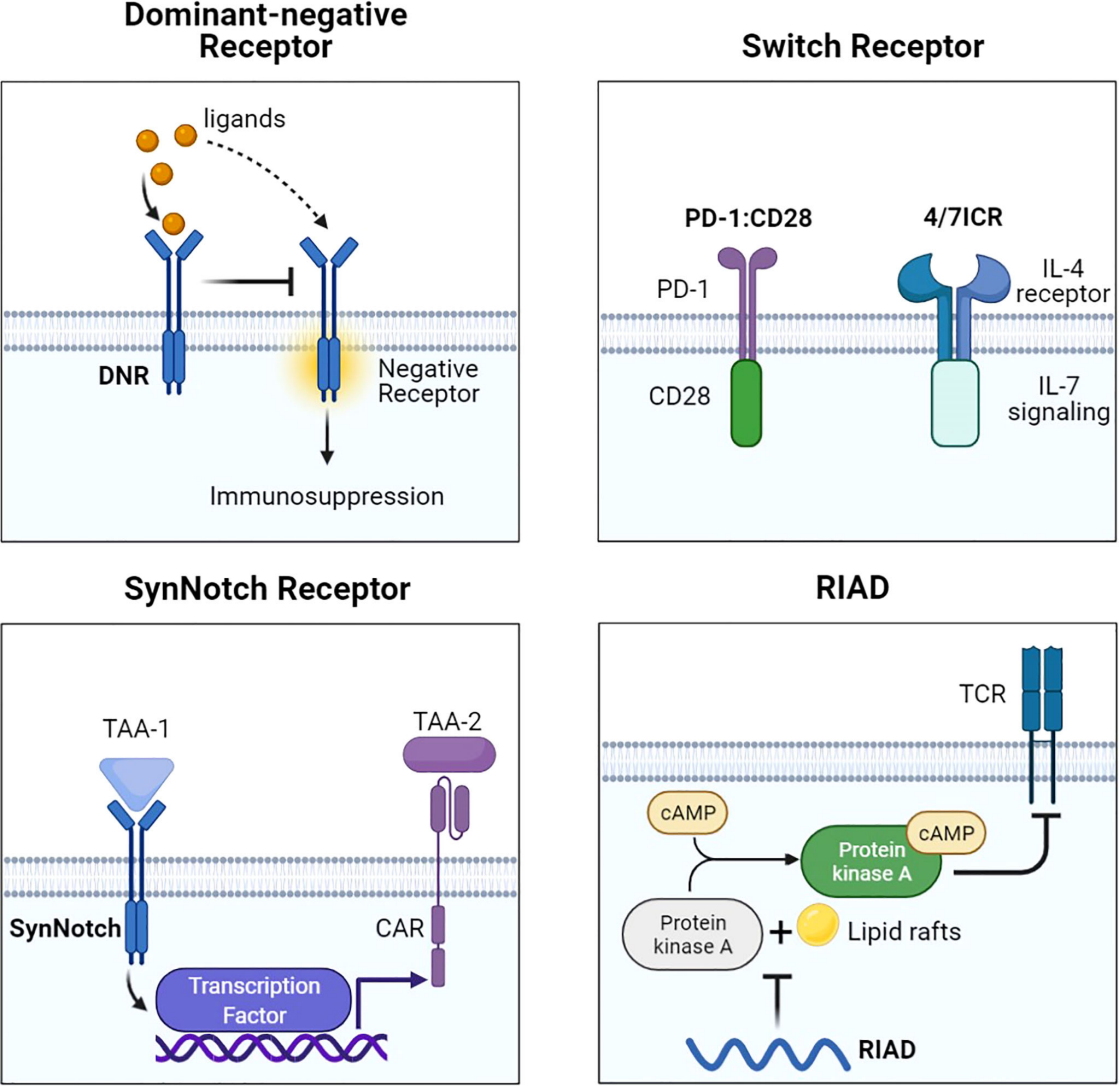
Representative designs of CARs with different receptors and modifications. Diagrams of CAR with different receptors and modifications. DNR avoided the suppressive signal by competing with negative receptors such as PD-1 and TGF-βR. Switch receptors converted the suppressive signal into activation signaling. SynNotch could increase antigen specificity by recognizing another TAA. RIAD inhibited the combination of PKA and cAMP to protect TCR. Figure created using BioRender.com.

**Figure 4:**
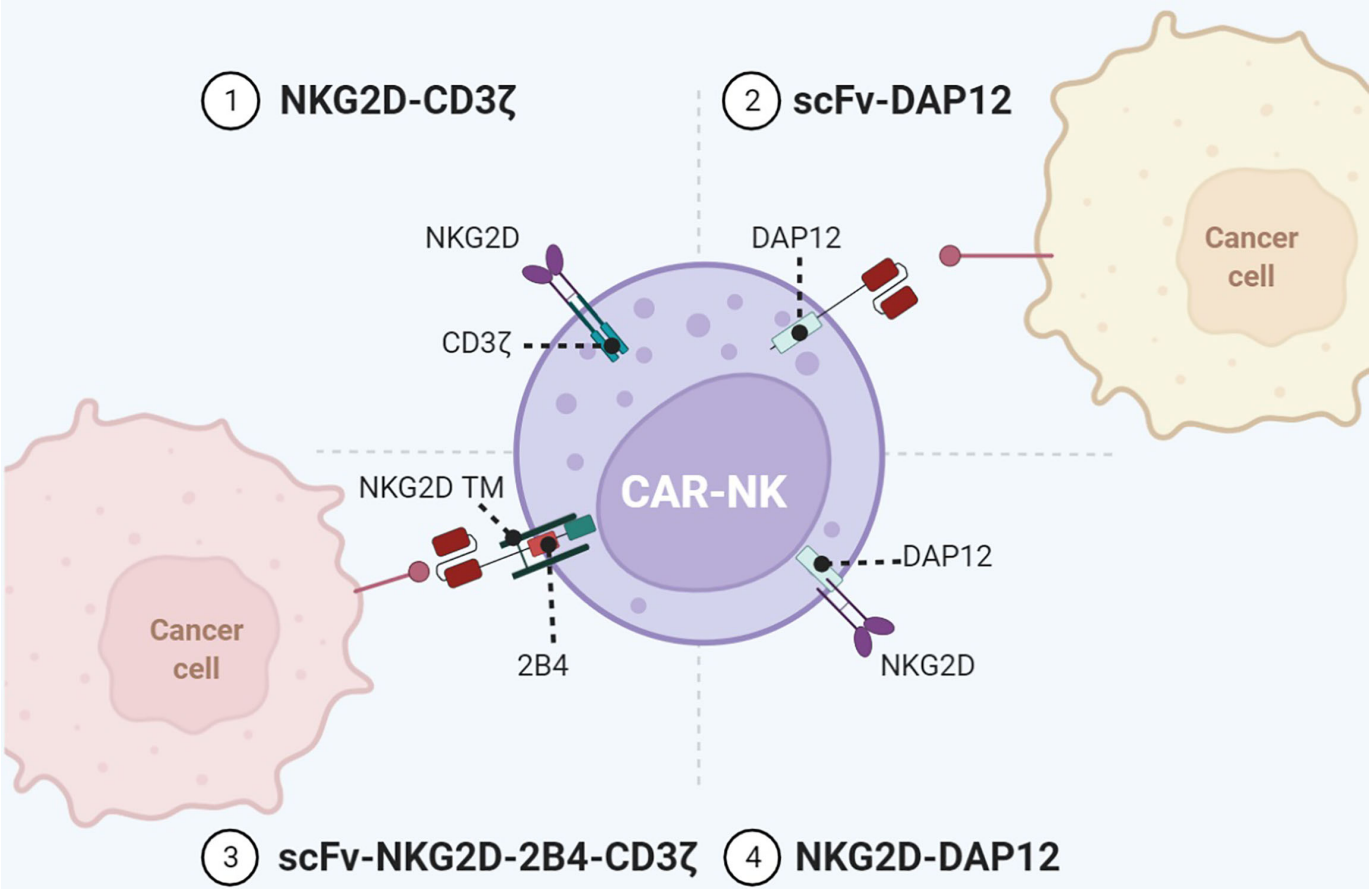
Representative designs of CARs with NK cell-associated repertoires. Diagrams of CAR-NK using NKG2D or DAP12 for immune activity. Co-expression of NK cell-associated receptors/adaptors such as NKG2D, KIR, TREM, DAP12, and 2B4 in the configuration of CARs enables a wider range of targets and enhanced antitumor immunity. Figure created using BioRender.com.

**Table 1:** CARs modified by NK cell receptors in solid tumors.

Target	Co-stimulatory domain	Activation domain	Cell type	Malignancy	Reference
NKG2D ligands	4-1BB	DAP12	T	Colorectal cancer/ovarian cancer models	(34)
NKG2D ligands	–	CD3ζ	NK	Neuroblastoma models	(35)
NKG2D ligands	–	DAP12	NK	Colorectal cancer patients	(36)
PSCA	–	DAP12	NK	Prostate cancer models	(37)
EGFRvIII	–	DAP12	NK	Glioblastoma models	(38)
	TM				
Mesothelin	KIR2DS2	DAP12	T	Mesothelioma models	(39)
Mesothelin	TREM1/KIR2DS2	DAP12	T	Mesothelioma models	(40)
Mesothelin	KIR2DS2	DAP12	T	Mesothelioma models	(41)
Mesothelin	NKG2D	CD244 (2B4)	NK	Ovarian cancer models	(42)

**Table 2:** CARs with different receptors and modifications in solid tumors.

Target	Receptor	Co-stimulatory domain	Activation domain	Cell type	Malignancy	Reference
	TCR					
NY-ESO-1/HLA-A2 complex	T1 scFv/T1 scFv with one mutation	CD28	CD3ζ	T	Melanoma models	(43)
gp100/HLA-A2 complex	GPA7	CD28	CD3ζ	T	Melanoma models	(44)
	TAC					
HER2	UCHT1/OKT3/L2K/F6A	–	CD3	T	Ovarian cancer models	(45)
	TRuC					
IL-13Rα2	TCRα/β, CD3γ/δ/ϵ	−	CD3	T	Glioblastoma models	(46)
	STAR					
EGFR	TCRαβ with cysteine mutations	–	CD3	T	Epidermis/brain/liver cancer models	(47)
	DNR					
Mesothelin	PD-1 DNR	CD28/4-1BB	CD3ζ	T	Mesothelioma models	(48)
PSMA	TGF-β DNR	4-1BB	CD3ζ	T	Prostate cancer models	(49)
–	TGF-β DNR	–	DAP12/SynNotch	NK	Neuroblastoma models	(50)
	Switch receptor					
Mesothelin/PSCA	PD-1/CD28	4-1BB	CD3ζ	T	Pleural mesothelioma/pancreatic carcinoma (PC) models	(51)
MUC1	4/7ICR	4-1BB	CD3ζ	T	Breast cancer models	(52)
PSCA	4/7ICR	CD28	CD3ζ	T	PC models	(53)
	SynNotch					
ROR1	EpCAM/B7-H3	4-1BB	CD3ζ	T	Breast cancer models	(54)
GD2	B7H3	4-1BB	CD3ζ	T	Neuroblastoma models	(55)
Mesothelin	RIAD	4-1BB	CD3ζ	T	Mesothelioma models	(56)

### T-Cell Receptor

Conventional TCR consists of α and β chains that combine with peptide-MHC ligands and cooperate with CD3 signaling, which has three dimers: ϵ/γ, ϵ/δ, and ζ/ζ (57). CD3 subunits have a total of 10 ITAMs that accept up to 20 tyrosine phosphates upon activation (58). On the one hand, TCR can be applied for antigen recognition. Compared with scFv mainly targeting membrane proteins, which account for about 30% of whole proteins (59), TCR could target almost all peptides *via* the MHC of antigen-presenting cells (APCs) (60). Transducing distinctive TCRs is a promising strategy for recognizing intracellular antigens. TCR–CAR composed of TCR α/β, CD28, and CD3ζ was designed and showed antitumor efficacy (61). Furthermore, antibodies with structural similarities to TCRs can also bind to HLA/peptide complexes. TCR-like CAR could guide T cells to eradicate xenograft melanoma (44). However, the excessive affinity of TCR may reversely reduce the specificity of CAR. Maus et al. developed a 2G CAR with the scFv of the high-affinity antibody (T1) and found that mutations of T1 scFv downregulated the avidity but upregulated the specificity and infiltration (43).

On the other hand, TCR has been exploited for CD3 signaling activation. Compared with CD3ζ containing three ITAMs, the TCR–CD3 complex can activate 10 ITAMs in total. Representative designs in this field include the T-cell antigen coupler (TAC), the TCR fusion constructs (TRuCs), the antibody-TCR (AbTCR), and the synthetic TCR antigen receptor (STAR) (**Figure 2**). TAC contained three domains, namely, an antigen-binding scFv, an anti-CD3 scFv, and a co-receptor domain (45). TAC could recruit the TCR–CD3 complex *via* the scFv targeting CD3. TAC-T cells manifested greater infiltration in solid tumors and less expansion in healthy tissues than 1G/2G CAR-T. The TAC platform without exogenous ITAMs could circumvent the tonic signaling. By fusing scFvs to extracellular N-termini of TCR subunits (TCRα/β, CD3γ/δ/ϵ), TRuCs were developed to activate CD3 in an MHC-independent manner (46). TRuC-T demonstrated either superior or equivalent tumor killing to CAR-T and released significantly less cytokines. AbTCR linked the Fab domain to γ and δ chains of TCR and avoided the mispairing with the endogenous αβTCR (62). AbTCR-T showed similar cytotoxicity with 2G CAR-T but less exhausted phenotype in CD19-expressing models. STAR is a double-chain TCR-based receptor with VH and VL, constant regions of TCR (TCR-Cα/β), and endogenous CD3 signaling machinery (47). In multiple tumor models, STAR-T exhibited similar persistence with 4-1BB-CAR-T and superior or equal antitumor effects compared with CD28-CAR-T.

### NK Cell Receptor

In addition to TCR, activation receptors on NK cells have also been incorporated into CAR to activate T cells. Among them, the type II transmembrane-anchored C-type lectin-like protein NKG2D is a key activation receptor of NK cells. NKG2D is not only located on NK cells but also presented on CD8^+^ αβT cells, γδ T cells, and NKT cells (63). NKG2D recognizes eight stress-induced ligands within two families: MHC class I chain-related proteins (MICA and MICB) and UL16-binding proteins (ULBP1–6) (64). These NKG2D ligands are rarely present on the normal cell surface but are upregulated upon DNA damage, hypoxia, infection, and transformation of cells (40). Since NKG2D ligands are widely distributed in various malignancies, NKG2D-CARs are capable of targeting a broad range of tumors. NKG2D-based CARs comprise either the full-length NKG2D receptor (aa 1–216) or the NKG2D ectodomain (aa 83–216) and contain the cytoplasmic tail of CD3ζ. By recognizing antigens *via* NKG2D, these CAR-T cells eradicate tumors in preclinical animal models (34). In a neuroblastoma xenograft model, NKG2D/CD3ζ-NK cells not only eliminated intratumoral myeloid-derived suppressor cells (MDSCs) with excessive NKG2D ligands but also guided GD2-CAR-T cells to tumor sites (35). It is noteworthy that activated T cells themselves also expressed ligands of NKG2D. NKG2D-CD3ζ-T cells without physiologic inhibitory receptors might encounter fratricide or on-target/off-tumor effects. In contrast, NKG2D-CD3ζ-NK cells merely targeted autologous MDSCs and did not attack NKG2D ligand-expressing T cells.

NKG2D merely has a short cytoplasmic tail, hardly initiating intracellular signaling. Specialized adaptors are indispensable for transmitting activation signaling received by NKG2D, especially the DAP10 in human and DAP12 in mouse. DAP10 can contribute to DAP12-dependent PI3K activation (25). In addition to CD3ζ, DAP12 can be another choice for activation upon NKG2D recognizing ligands. Despite that the NKG2D/DAP12 axis is absent in human immune cells, the artificial NKG2D/DAP12 structure exhibits considerable immunocompetence (**Figure 4**). DAP12 is a short 12-kDa transmembrane (TM) protein of 113 aa expressed on the surface of a broad range of hematopoietic cells, harboring a single ITAM in the 48-aa cytoplasmic domain (65). The phosphorylation of ITAM activates the downstream cytoplasmic ZAP70 tyrosine kinases to eventually produce cytokine (66). DAP12 ITAM also contains an immunoreceptor tyrosine-based inhibitory motif (ITIM), which is not present in CD3ζ (67). This property makes DAP12 adjust its reaction in response to stimuli with differential strength to avoid CAR tonic signaling. Producing NKG2D/DAP12-CAR-NK by mRNA electroporation could treat patients with metastatic colorectal cancer (36). In two patients with malignant ascites, the number of tumor cells in ascites was significantly reduced after infusion of CAR-NK. The patient treated with ultrasound-guided percutaneous injection of CAR-NK in the liver acquired a complete metabolic response at the injection site. A phase 1 trial of NKG2D/DAP12-CAR-NK in metastatic colorectal cancer patients is ongoing (NCT05213195). Temme’s lab has developed two DAP12-CAR-NK cells with scFvs targeting PSCA or EGFRvIII (37, 38). They reported that co-stimulatory domains are unnecessary for DAP12-mediated NK cell activation.

Moreover, T cells can employ NKG2D/DAP12 as well. Chronic CD3ζ activation may induce tonic signaling and cause impaired expansion of CAR-T, while DAP12 enables non-impaired production of CAR-T because of the ITIM motif. Compared with NKG2D/CD3ζ-CAR-T, NKG2D/DAP12-CAR-T secreted less interferon (IFN)-γ, TNF-α, and IL-2 during tumor cell lysis and mitigated the risk of CRS (34). Considering that the ITIM motif can preclude tonic signaling (25), Epstein et al. designed CAR with DAP signaling domains to prevent CAR-T exhaustion (68). Excluding NKG2D, DAP12 can associate with other receptors to initiate activation. In the lymphoid lineage like NK cells, DAP12 binds to receptors such as NKG2C, NKp44, the short-tailed KIR3DS1, and KIR2DS1/2/5 (37). Killer immunoglobulin-like receptors (KIRs) are transmembrane glycoproteins detecting MHC class I molecules. KIR genes can be translated into inhibitory receptors (2DL and 3DL) or activating receptors (2DS, 3DS, and 2DL4) with two or three C2-type Ig-like extracellular domains. In the myeloid lineage, like macrophages and granulocytes, DAP12 is coupled with a triggering receptor expressed on myeloid cell members (TREM) or myeloid DAP12-associating lectin 1 (MDL1) (69, 70). Investigators have attempted to fuse scFv to KIR2DS2/TREM-1 TM and use DAP12 for activation (40). Compared with CD3ζ-CAR-T, KIR2DS2/TREM-1-CAR-T exhibited superior antitumor activity despite lower IL-2 production. Their enhanced antitumor activity was associated with better maintenance of CAR expression in TILs (39). Our team further designed KIR2DS2/DAP12-CAR-T cells with the presence of 4-1BB (41). DAP12 accompanied by 4-1BB made T cells express more central memory phenotype and less PD-1. Our phase I clinical trial found that CD19-KIRS2/DAP12-4-1BB CAR-T cells were safe and efficient in treating relapsed/refractory B-ALL patients.

### DNR

To counteract the PD-1/PD-L1 axis, PD-1 dominant-negative receptor (DNR) saturating PD-L1 was designed to compete with endogenous PD-1. DNR only contained an extracellular receptor and a CD8 TM. CAR-T with PD-1 DNR demonstrated improved efficacy (48). In addition to PD-1, transforming growth factor β (TGF-β) secreted by solid tumors and received by the TGF-βRII/TGF-βRI heterodimer complex can induce an immunosuppressive milieu. The dominant-negative TGF-βRII, which lacks the intracellular domain, can be employed for CAR. TGF-β-insensitive CAR-T had a striking proliferative advantage over wild-type CAR-T in prostate cancer models (49). TGF-β DNR CAR-T showed a gene profile associated with cell cycle progression and cell division and a cytokine profile including the T helper (Th) 2 phenotype (IL-4, IL-5, and IL-13) and the Th1 phenotype (IL-2 and IFN-γ). TGF-β can phosphorylate Smads 2 and 3 to impair the expression of NK receptors. TGF-β DNR was also tailored with DAP12 or a synthetic Notch-like receptor to protect NK cells in neuroblastoma (50).

### Switch receptor

External inhibitory receptors with cytoplasmic activation domains are called “switch receptors” (51). For example, a PD-1/CD28 switch receptor was incorporated into mesothelin or PSCA CAR-T and enhanced tumor eradication. The PD-1 switch receptor was more helpful for CAR-T cells than current PD-1 inhibitors. Among the six diffuse large B-cell lymphoma patients treated with CD19-PD-1/CD28-CAR-T, half achieved complete remissions (71). Some cytokines in the TME, such as IL-4, IL-10, and TGF-β, also induce suppressive signals. The MUC1-CAR with the cytokine switch receptor (4/7ICR), which consists of the IL-4 receptor extracellular domain and the IL-7 intracellular signaling domain, efficiently suppressed tumor growth in breast cancer models and did not show markers of exhaustion (52). In the IL-4-rich pancreatic tumor milieu, PSCA-4/7ICR-CAR-T also significantly repressed the tumor (53).

### SynNotch receptor

The synthetic notch (SynNotch) system is a gating strategy to minimize the off-tumor toxicity of CAR-T. The SynNotch receptor comprises an extracellular antigen-recognition domain. Matching ligations lead to the release of intracellular transcription factors into the nucleus to activate the secondary CAR gene (72). Intermittent gate-dependent expression of the second CAR relieves tonic signaling and improves metabolic fitness. SynNotch receptors targeting B7-H3 or EpCAM were used to activate ROR1-CAR (54). Choosing GD2 as the gatekeeper for B7H3-CAR also showed improved metabolic plasticity, high specificity, and low toxicity in neuroblastoma (55).

### RIAD

Another choice to overcome CAR-T limitations in solid tumors is to maintain TCR-induced T-cell proliferation and cytotoxic ability (73). Protein kinase A (PKA) on the immune synapse phosphorylates key molecules to inhibit the TCR signaling cascade. PKA is activated through a cyclic AMP (cAMP)-dependent manner. It is necessary for PKA to be tethered to lipid rafts in close proximity to the cAMP-generating enzyme adenylyl cyclase. One peptide, called “regulatory subunit I anchoring disruptor” (RIAD), could displace PKA from lipid rafts (74). Newick and colleagues designed RIAD-CAR to protect TCR from cAMP/PKA-mediated immunosuppression (56). Mesothelin-RIAD-CAR-T showed increased TCR signaling and infiltrated into the mesothelioma to secrete more cytokines.

## Developments of intracellular signaling

Physiological T-cell activation requires three signals (75). Signal 1 (activation) is regulated by CD3ζ upon antigen recognition. Signal 2 (co-stimulation) originates from co-stimulatory molecules supporting CD3ζ. Signal 3 is secreted by cytokines, promoting T-cell proliferation, differentiation, and development. Transducing signals suitable for tumoricidal cytotoxicity could expand advantages for CAR-T in solid tumors (**Table 3**).

**Table 3:** CAR with different stimulation domains in solid tumors.

Target	Co-stimulatory domain	Activation domain	Cell type	Malignancy	Reference
B7H3	4-1BBL^+^CD28	CD3ζ	T	Metastatic osteosarcoma/lung cancer models	(76)
Mesothelin	TLR2^+^CD28	CD3ζ	T	Mesothelin expressing tumor cells	(77)
HER2	MyD88/CD40/FKBP12^+^CD28	CD3ζ	T	Osteosarcoma models	(78)
PSCA/GD2/CD123	MyD88/CD40/FKBP12^+^CD28	CD3ζ	T	PC/leukemia models	(79)
HER2	MyD88/CD40/FKBP12	CD3ζ	T	HER2 expressing solid cancers	(80)
EphA2	MyD88/CD40	CD3ζ	T	Osteosarcoma cell lines	(81)
EGFRvIII	ICOS	CD3ζ	T	Glioma cells	(82)
Mesothelin	ICOS	CD3ζ	CD4^+^ T	Mesothelioma models	(83)
Mesothelin	ICOS^+^4-1BB	CD3ζ	T	PC models	(32)
FR	CD27	CD3ζ	T	Ovarian cancer cells	(84)
Trophoblast cell surface antigen 2 (Trop2)	CD27	CD3ζ	T	Breast cancer models	(85)
GD2	4-1BB^+^IL-12/18	CD3ζ	T	Patient-derived primary glioblastoma cells	(86)
GPC3	CD28^+^IL-12	CD3ζ	T	GPC3 expressing hepatocellular carcinoma models	(87)
CEA	CD28^+^IL-18	CD3ζ	T	Advanced PC and lung tumor models	(88)
B7H3	4-1BB^+^IL12β p40	CD3ζ	T	Pancreatic ductal adenocarcinoma (PDAC) models	(89)
EphA2/HER2	CD28^+^GM18	CD3ζ	T	EphA2/HER2 expressing models	(90)
GD2/EphA2	4-1BB^+^C7R	CD3ζ	T	Neuroblastoma/glioblastoma models	(91)
GPC3/mesothelin	CD28^+^IL-7^+^CCL19	CD3ζ	T	Hepatocellular carcinoma (HCC)/PC models, advanced HCC/PC/ovarian carcinoma patients	(92, 93)

### Modification of CD3ζ

The location and number of ITAMs are tightly associated with CAR-T functions. James and colleagues demonstrated that deleting a CD3ζ ITAM helped CD19-CAR-T to focus on CD19 highly expressing cells and ignore normal cells (94). Sadelain and colleagues found that a single functional ITAM was sufficient for antitumor efficacy and superior to the natural triple-ITAM-containing CD3ζ chain in a CD28-based CAR-T (26). The single ITAM configuration prolonged the lifetime of CAR-T and balanced the replicative capacity of long-lived memory cells. Furthermore, ITAM showed a stronger signal transduction ability when closer to TM. However, increasing the number of ITAMs in 4-1BB-based CAR-T was beneficial for recognizing low antigen density-expressing target cells (95). Thus, the function of each ITAM might be qualitatively different. Selecting the optimal ITAM(s) for different CAR signaling domains is a feasible strategy to promote solid tumor eradication.

### Selection of Co-Stimulatory Domains

**Figure 5:**
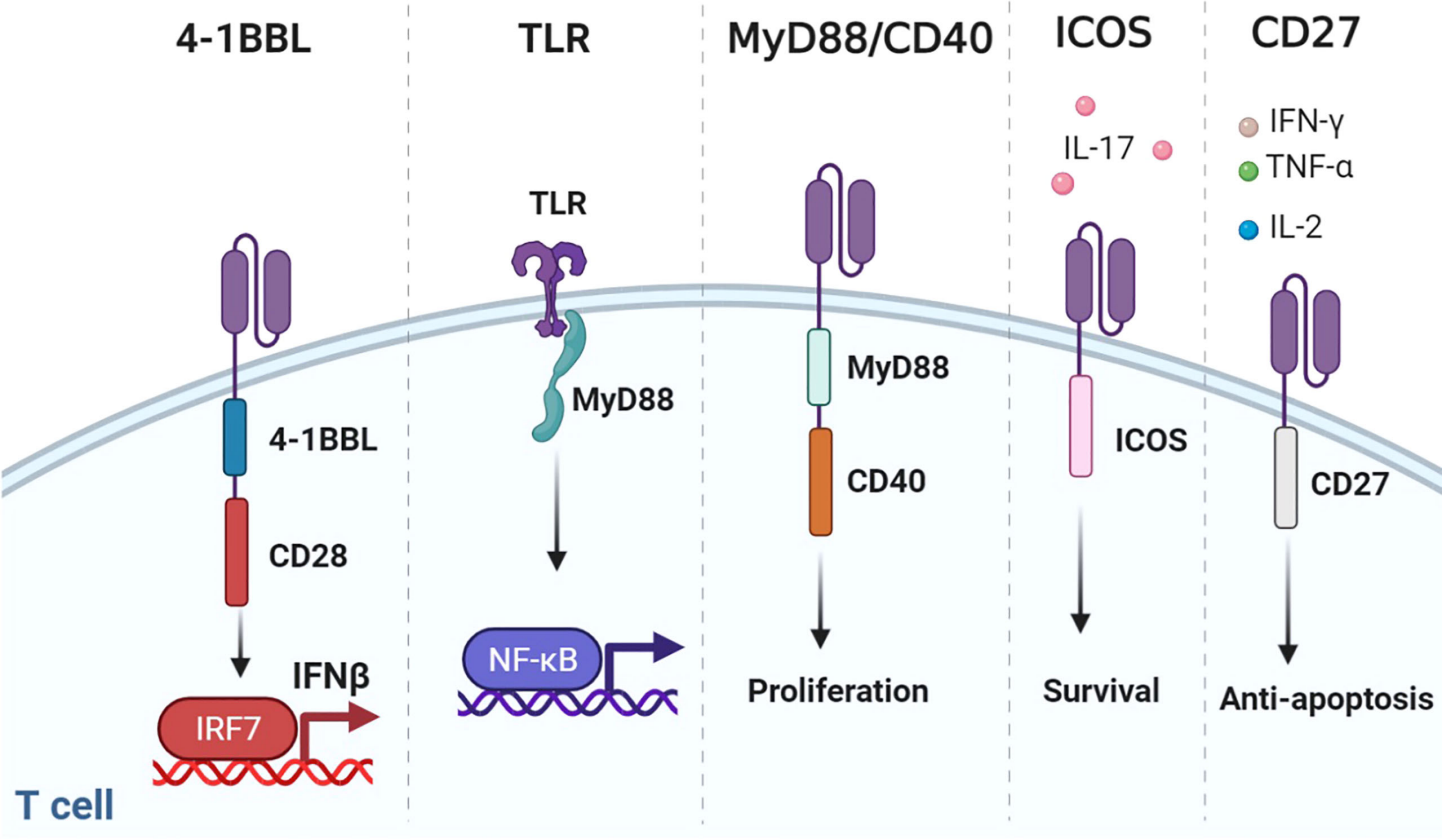
Representative designs of CARs with co-stimulatory domains. Diagrams of CARs with co-stimulatory domains. 1) Co-expressing 4-1BBL and CD28 could recruit the host immune response against the tumor through the IRF7/IFN-β pathway. 2) TLR could combine MyD88 to activate NF-κB. 3) MyD88/CD40 promoted T-cell proliferation. 4) ICOS increased the production of IL-17 and enhanced the survival of T cells. 5) CD27-based CAR-T cells secreted Th1 cytokines (IFN-γ, TNF-α, and IL-2) and were resistant to apoptosis. Figure created using BioRender.com.

Co-stimulatory molecules can be divided into two groups. The first is basically expressed on resting antigen-naive T cells including CD27 and CD28. The other, including ICOS, 4-1BB, OX40, and GITR, is detectable after the formation of immune synapses (29). CD28 and/or TNFR family members (4-1BB and OX40) are usually chosen as co-stimulatory domains (96). Details of CD28 and 4-1BB have been reviewed elsewhere (97). Here, we presented other co-stimulators and their combinations (**Figure 5**).

### 4-BBL

Although CD28 and 4-1BB are the most common co-stimulatory molecules, changing their location or construct results in spatial and temporal differences. Considering that CD28 far away from the cell membrane would weaken the function of CAR, multiple attempts have been made to figure out the optimal design of 3G CARs (98, 99). Surprisingly, compared with 3G CAR incorporating 4-1BB, placing 4-1BB ligand (4-1BBL) on the surface endowed CAR-T with superior function in solid tumors (76). This may have resulted from the trimeric conformation of activated 4-1BB distinct from the dimeric CAR design. Co-expressing 4-1BBL and CD28 could recruit the host immune response against the tumor through the IRF7/IFN-β pathway (100). The safety and efficacy of CD28-CAR-T with surface 4-1BBL were evaluated in a clinical trial for adults with CD19-positive malignancies (NCT03085173).

### TLR

Toll-like receptors (TLRs) are innate immune receptors on activated T cells for co-stimulation (101). TLR2 signaling can induce the secretion of T-bet protein, IFN-γ, perforin, and granzyme B through the mTOR pathway (102). TLR2 potentiated the antitumor efficacy of CAR-T without increased CRS (77). Genes upregulated by TLR2 co-stimulation were mainly associated with cell adhesion, synaptic transmission, and migration. TLR2 can form weak homodimers to combine MyD88 (103), which activates NF-κB and PI3K/AKT signaling and provides anti-apoptotic signals in T cells (104).

### MyD88/CD40

The MyD88/IRF7 complex is critical for IFN production (105). Therefore, investigators exploited MyD88 as the co-stimulatory domain. An artificial design consisting of MyD88, CD40, and FKBP12 could be used for CAR-T (78, 79). CD40 is a cell-surface receptor for T-cell intrinsic activation, differentiation, memory formation, and exhaustion resistance (106). Researchers utilized the small molecule ligand rimiducid (Rim) to dimerize MyD88/CD40 by binding to FKBP12. Dropping Rim induced IL-2 secretion and promoted proliferation. On the contrary, withholding Rim effectively eliminated residual CAR-T. Moreover, CAR-T directly transduced with MyD88/CD40 expressed higher levels of MYB and FOXM1 at baseline and remained at a less differentiated state after stimulation than CD28- or 4-1BB-CAR-T (81).

### ICOS

ICOS is a member of the CD28 family. ICOS activates PI3K/AKT signaling more potently than CD28. ICOS-based CAR-CD4^+^ T cells demonstrated a Th1/T17 polarization, producing more IL-17A and less IL-2, and enhanced the survival of CD28 or 4-1BB-based CAR-CD8^+^ T cells (32, 83). The combination of ICOS and 4-1BB as the 3G CAR showed synergistic effects on antitumor activity. Placing co-stimulatory domains proximal to the cell membrane could reduce CAR expression on the cell surface to avoid tonic signaling.

### CD27

CD27 is a member of the tumor necrosis factor receptor superfamily, constitutively expressed on immune cells (84). CD27-based CAR-T cells secreted Th1 cytokines (IFN-γ, TNF-α, and IL-2) and exhibited stronger toxicity and more Bcl-XL, an anti-apoptotic protein of the Bcl-2 family (107). Increased IL-7Rα and decreased PD-1 were presented on CD27-based CAR-T compared with CD28-based CAR-T, which indicated that CD27 could prevent T-cell exhaustion (85).

### Transduction of Signal of Cytokines and Chemokines

**Figure 6:**
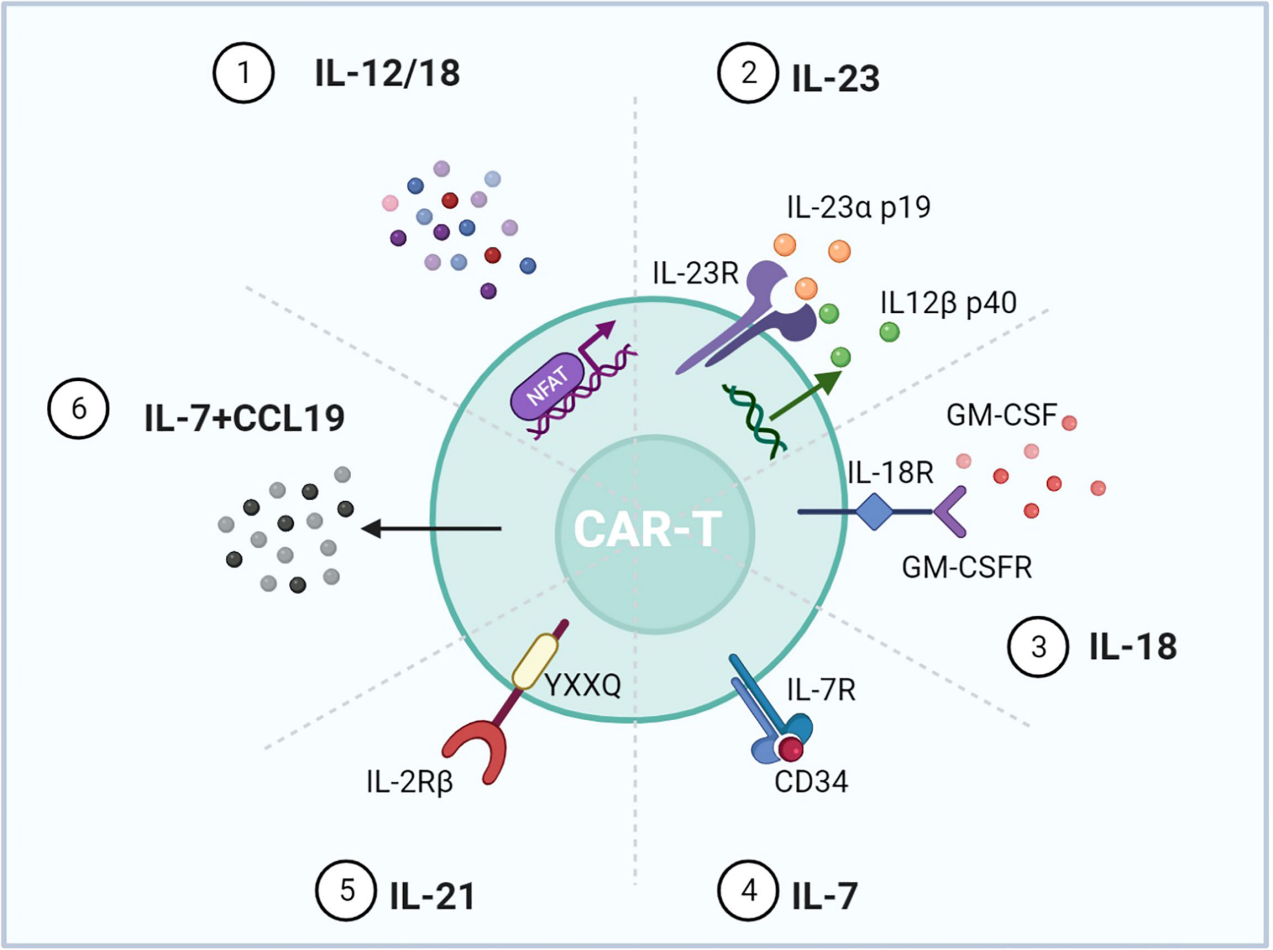
Representative designs of CARs with cytokines and chemokines. Diagrams of CAR mediating cytokine and chemokine expression upon T-cell activation. 1) NFAT activated upon antigen recognition was transduced into T cells to regulate IL-12/18. 2) IL-12β p40 was overexpressed for IL-23 production accompanied by intrinsic IL-23R and IL-23α p19. 3) The GM-CSF receptor sensing GM-CSF upon T-cell activation was incorporated with the IL-18 receptor. 4) The IL-7 receptor was enough for the downstream STAT5 activation, and CD34 blocked the effect of exogenous IL-7. 5) IL-2Rβ with the YXXQ motif could mimic the effect of IL-21. 6) Expressing IL-7 and CCL19 could promote CAR-T migration and recruit DCs and T cells into the tumor. Figure created using BioRender.com.

4G CAR-T is characterized by using signals derived from immunostimulatory cytokines, known as T cells redirected for universal cytokine-mediated killing (TRUCK) (6). Forced expression of cytokine genes, such as IL-12 and IL-15, can improve the persistence and antitumor effects of CAR-T but increase the risks of serious adverse events (108, 109). To control cytokine signaling only upon antigen engagement, different strategies were used to regulate the cytokine expression (**Figure 6**). Firstly, scientists transduced T cells with CAR and inducible cytokine expression through nuclear factor of activated T cells (NFAT)-responsive promoters (86, 87). IL-12 or IL-18 expression was ideally induced upon tumor antigen recognition. Armored inducible expression of cytokines could facilitate CAR-T function with fewer potential side effects. Inducible IL-18 could further accumulate FoxO1 transcription factor and alleviate T-bet to sustain a cytolytic phenotype. IL-18 CAR-T could recruit M1 macrophages and NKG2D^+^ NK cells and drive away immune-suppressive cells (88). Moreover, IL-23 is a STAT3-activating cytokine and consists of IL-23α p19 and IL-12β p40 subunits. Upon TCR stimulation, T cells upregulate the expression of IL-23 receptors and the IL-23α p19 excluding the p40 subunit. Therefore, CAR-T with p40 (p40-Td CAR-T) could couple the release and activity of IL-23 with T-cell activation (89). IL-23 produced by p40-Td cells worked mainly through an autocrine mechanism with limited effects on bystander cells. Additionally, GM-CSF, a cytokine invariably expressed after CAR-T activation, provides another choice for inducible cytokine expression (90). A chimeric cytokine receptor (GM18) was constructed by combining the extracellular domains of the GM-CSF receptor with the TM and signaling domains of the IL-18 receptor. GM18 created a functional loop to secrete IL-18 upon antigen exposure and activated the MyD88 signaling. On the other side, CAR-T with cytokine receptors was developed. Enlightened by the report that constitutively active IL-7 receptors without ligands are sufficient for STAT5 activation, Shum et al. fused a constitutively active IL-7 receptor variant with ectodomains derived from CD34, which blocked the external IL-7, to construct C7R (91). C7R co-expression in CAR-T exerted a broad anti-apoptotic influence by upregulating BCL2 and downregulating FAS and CASP8, consistent with the role of IL-7. C7R-CAR-T showed superior antitumor activities in multiple models. Moreover, IL-2 induces STAT5 activation through tyrosine residues within the IL-2 receptor β-chain (IL-2Rβ), while IL-21 preferentially activates STAT3 through its association motif YXXQ within the IL-21 receptor (110). Investigators have incorporated the truncated cytoplasmic domain of IL-2Rβ and a STAT3-binding YXXQ motif into CAR-T targeting CD19 (111). Compared with a CD28 or 4-1BB domain alone, this kind of CAR-T cells showed antigen-dependent JAK–STAT3/5 pathway activation and gene expression profiles analogous to be triggered by IL-21, which promoted their proliferation and prevented terminal differentiation.

In addition, chemokines are a kind of small molecule cytokine that can induce chemotaxis. Since solid malignancies are difficult to penetrate, expressing chemokines can attract immune cells to traffic into the tumor. IL-7 and CCL19 are essential for the formation and maintenance of the T-cell zone in lymphoid organs, where both T cells and dendritic cells (DCs) are recruited from the periphery. CAR-T cells expressing IL-7 and CCL19 were designed (7 × 19 CAR-T) (92). IL-7 and CCL19 expression has been identified to improve T-cell infiltration and CAR-T survival in mouse tumors. Compared to conventional CAR-T, 7 × 19 CAR-T showed superior antitumor activity and recruited DCs and T cells. A phase 1 clinical trial was conducted to evaluate the clinical potential of 7 × 19 CAR-T (NCT03198546) (93).

## Developments of carrier immune cells

Beyond conventional CD4^+^/CD8^+^ T cells, immune cells involved in the innate immune system can carry CAR. Based on their specific constructs, they are with favorable factors to circumvent GvHD and show potent cell-killing abilities (**Table 4**).

**Table 4:** CAR carried by different immune cells in solid tumors.

Target	Co-stimulatory domain	Activation domain	Cell type	Malignancy	Reference
HER2	CD28	CD3ζ	NK	Breast cancer/renal cell carcinoma models	(112)
NKG2D ligands	–	CD3ζ	NK	Neuroblastoma models	(35)
ROBO1	4-1BB	CD3ζ	NK-92	A PDAC patient	(113)
Mesothelin	2B4/DAP10/DAP12/4-1BB/CD28	CD3ζ	iPSC-derived NK	Ovarian cancer models	(42)
GPC3	CD28^+^4-1BB	CD3ζ	iPSC-derived NK	Ovarian cancer models	(114)
CEA	–	CD64	Monocytes	Gastric carcinoma models	(115)
Mesothelin/HER2	–	CD3ζ	Macrophages	Ovarian cancer models	(116)
Mesothelin	CD86	FcγRI	iPSC-derived macrophages	Mesothelin-expressing models	(117)
MCSP	CD28	CD3ζ	γδ T	Melanoma cells	(118)
GD2	CD28	CD3ζ	γδ T	Neuroblastoma cells	(119)
GD2	CD28/4-1BB/CD28^+^4-1BB	CD3ζ	iNKT	Neuroblastoma models and patients	(120, 121)
CD44v6	CD28	CD3ζ	CIK	Soft tissue sarcoma	(122)
FRα	–/CD28/CD28^+^4-1BB	CD3ζ	CIK	Ovarian cancer models	(123)
5T4	CD28	CD3ζ	CIK	Nasopharyngeal carcinoma cell lines	(124)
GD2	–	CD3ζ	EBV-specific T	Neuroblastoma patients	(125)
HER2	CD28	CD3ζ	EBV-specific T	HER2-expressing models	(126)
GD2	–	CD3ζ	CMV-specific T	GD2-expressing models	(127)
HER2	CD28	CD3ζ	Virus-specific T cells	HER2-positive progressive glioblastoma patients	(128)
Mesothelin	–	CD3ζ	CD26^high^ CD4^+^ T	Mesothelin-expressing models	(129)

### NK Cells

NK cells without TCR-like molecules are distinct from T cells and of paramount importance in the innate immune system by performing MHC-unrestricted cell killing. NK cells express a repertoire of inhibitory and activating receptors and related adaptors, including natural cytotoxic receptors such as NKG2D, CD16 (FcγRIIIa), FasL, and TRAIL, and co-stimulatory receptors such as LFA-1, 4-1BB, and 2B4 (130). When KIRs on the NK cell surface fail to engage with their cognate HLA, cell lysis is activated. This phenomenon is summarized as the “missing self” mechanism (131). The NK cell-based therapy has superiority in the microenvironment with downregulated HLA. Inhibitory receptors of NK cells can combine HLA class I molecules on autologous normal cells to relieve cytotoxic activity, avoiding CRS or neurotoxicity (132). Furthermore, NK cells as allogeneic effectors do not need to be collected from a patient or a specific HLA-matched donor. NK cells can be acquired from umbilical cord blood (UCB) to generate “off-the-shelf” products. With over 500,000 validated banked UCB units worldwide, the source of adoptive NK cells is available (133). NK cells show cytotoxicity advantages over T cells in CAR-driven immunotherapy (134). CD3ζ modification made CAR-NK cells resistant to TGF-β, which inhibits endogenous NK cells by decreasing DAP10 transcription (35, 135). Incorporating CD19-CAR-NK cells with IL-15 could further promote NK cell expansion and persistence. Of the 11 patients with relapsed/refractory CD19-positive cancers, 7 had a complete remission after the treatment of CD19-CAR-NK cells with IL-15 (136).

In solid tumors, a series of trials employing CAR-NK are ongoing (NCT03941457, NCT05194709, NCT03940820) (113, 137). Intriguingly, CAR-expressing lymphoid progenitors were prone to differentiate into cells with NK cell receptors that are called CAR-induced killer cells (CARiK) by inhibiting the transcription factor B-cell CLL/lymphoma 11B (BCL11B) and NOTCH1. Allogeneic CARiK showed increased survival without GvHD, suggesting the potential of CARiK for anticancer immunotherapy (138). Moreover, CAR-expressing induced pluripotent stem cell (iPSC)-derived NK cells with the TM domain of NKG2D showed significant antitumor cytotoxicity. Moreover, the co-stimulatory domain 2B4 instead of DAP10, DAP12, 4-1BB, or CD28 gave the greatest antitumor activity (42). Since 2B4 signaling is necessary for optimal NK cell function and beneficial for activation triggered by other NK cell receptors (139), 2B4 might be the most suitable candidate for the co-stimulatory domain of CAR-NK cells and more investigation is needed.

### Macrophages

Macrophages can serve as the platform for CAR (116). CAR macrophages (CAR-Ms) exhibited versatilities including tumor-specific phagocytosis, inflammatory cytokine production, polarization of bystander macrophages to the immunostimulatory M1 phenotype, and cross-presentation of the TAAs to bystander T cells. Resultantly, CAR-Ms could create a pro-inflammatory TME and promote antitumor T-cell activity. Additionally, iPSC−derived macrophages with the CAR consisting of the macrophage receptor FcγRI and CD19 or mesothelin scFvs demonstrated enhanced phagocytosis and immune activities (117).

### γδ T Cells

γδ T cells are T cells with γ/δ TCRs, constituting only 1%–5% of circulating lymphocytes but predominant in some epithelial sites, such as the intestine, reproductive organs, tongue, and skin (140). Similarly, γδ T cells could accumulate in the solid TME. αβ T cells exert antitumor effects in an MHC-dependent manner. In contrast, γδ T cells find their targets through innate-like pathways without the restriction of MHC. They sense small, non-peptide antigens called “phosphoantigens.” With the capability of migration toward tumors and cross-presentation of TAAs, γδ T cells are more beneficial for the adoptive T-cell therapy in solid tumors than αβ T cells. Furthermore, γδ T cells are less prone to exhaust or induce GvHD than αβ T cells (141, 142). Consequently, γδ T cells have the potential to become “off-the-shelf” cellular immunotherapy (143). Compared with genetically engineered conventional T cells, γδ T cells with the expression of TCR or CAR released reduced cytokines but presented the equivalent cytotoxicity in the melanoma (118). CAR-γδ T cells with co-stimulation and antigen-presentation molecules (CD86 and HLA-DR) were able to cross-present TAAs to other T cells in the TME (119). The abovementioned AbTCR combined the Fab domain with γ and δ chains. CD19-AbTCR-T cells showed similar cytotoxic activity with CD19^–^CD28/4-1BB CAR-T cells but with less cytokine and exhaustion (62). More specifically, there are two main subtypes of γδ T cells (**Table 5**) (151). The first is Vγ9Vδ2 T cells, whose TCR heterodimer is built by a γ chain with the Vγ9 segment and a δ chain with the variable segment Vδ2. Another is Vδ1^+^ T cells containing the Vδ1 segment. CAR-Vδ1^+^ cells maintained a T-naive phenotype, and CAR-Vδ2^+^ cells predominantly adopted an effector memory phenotype. CAR-Vδ1^+^ cells showed less exhausted phenotypes than their Vδ2^+^ counterparts (119).

**Table 5:** Differences between Vδ1^+^ and Vδ2^+^ T cells.

	Vδ1^+^	Vδ2^+^
Spatiotemporal heterogeneity	Resident in tissues (144) 20%–50% of the tissue-resident lymphoid compartment (145) Dominate during fetal development and childhood (146)	Resident in circulating peripheral blood 2%–5% of circulating CD3^+^ lymphocytes (147) Dominate during adulthood
Cytotoxicity	Higher than δ2^+^ T cells (144) Perforin, granzymes, IFN-γ, and TNF	Perforin, granzymes, IFN-γ, and TNF (145)
Durability	Resistant to activation-induced cell death (AICD) (148)	Sensitive to AICD
Receptors	Vδ1^+^ TCR, NKG2D, NKp30, NKp44, NKp46 (149)	Vγ9^+^Vδ2^+^ TCR, NKG2D, CD16, FasL, TRAIL, and DNAM-1 (150)

### iNKT Cells

NKT cells are a subset of T lymphocytes with NK cell surface markers. They are rare but powerful effector T cells. Chemokines secreted by tumor cells and tumor-associated macrophages (TAMs) can recruit NKT cells into solid tumors (152). The non-polymorphic, glycolipid-presenting HLA I-like molecule CD1d on B cells presents antigens for recognition of NKT cells (153). Reciprocally, NKT cells can eliminate TAMs (154). The monomorphic nature of CD1d and its interspecies conservation reduces the risk of GvHD. CD1d-restricted Vα24-invariant (type-I) NKT, also termed iNKT, whose infiltration in colon cancer is associated with a better prognosis, is one of the ideal candidates for CAR carrier (155). Compared with CAR-T cells, CAR-iNKT cells are superior as they can easily arrive at tumor sites, kill CD1d-positive TAMs, and avoid GvHD. In B-cell malignancies, CD19-CAR-iNKT cells exhibited superior proliferation and therapeutic effects than CAR-T cells. Upregulating CD1d expression could enhance the antitumor efficacy of CD19-CAR-iNKT cells (156). In particular, given that the co-stimulatory OX40L–OX40 axis is conducive for iNKT cell-mediated antitumor responses, the inclusion of OX40 in a 3G CAR configuration might be advantageous. In neuroblastoma, GD2-based CAR rendered iNKT cells with stronger cytotoxicity and persistence bypassing CD1d. Moreover, 4-1BB induced T helper 1-like polarization of NKT cells (120). Researchers further engineered GD2-CAR-iNKT cells with IL-15 and initiated a first-in-human CAR-NKT clinical trial (NCT03294954) (121). Co-expression of IL-15 could reduce exhaustion markers and improve tumor control. In addition, they reported using CD28 instead of 4-1BB as the co-stimulatory domain could avoid excessive activation-induced cell death. The interim analysis showed that the GD2-targeted CAR-NKT cells were well tolerated in three patients. One of the three patients achieved an objective response with regressing bone metastatic lesions (157).

### CIKs

Cytokine-induced killer cells (CIKs) are *ex-vivo*-expanded T lymphocytes with T-NK phenotype and potent MHC-independent antitumor ability regulated by the NKG2D receptor. Researchers aimed at combining the CAR specificity with the intrinsic tumor-killing ability of CIK cells to generate bipotential killers. CAR-CIKs have been investigated in solid tumor settings. CAR-CIK produced higher amounts of IL-6 and IFN-γ and enhanced killing activity compared to control CIK (122). The presence of 4-1BB or CD28 in CAR increased the production of cytokines, and the 3G CAR-CIKs showed significant proliferation and long-term inhibition on tumor growth (124, 158).

### Virus-Specific T Cells

Cytotoxic T lymphocytes (CTLs) specific for endogenous viral antigens such as Epstein–Barr virus (EBV) and cytomegalovirus (CMV), known as virus-specific T cells (VSTs), receive superior co-stimulation provided by professional APCs. Repeated stimulation with viral antigen gradually increases viral specificity and concomitantly depletes alloreactivity. Clinical application of donor virus-specific T cells indicates a low rate of GvHD (159). EBV-specific T-cell lines contain CD8^+^ cytotoxic T cells and antigen-specific CD4^+^ helper T cells, which control EBV latency by providing growth factors (160). The rapid expansion of EBV-specific T cells *in vivo* and their persistence in a lifelong functional state without further immunization make them attractive candidates for CAR vehicles. CD8^+^ T cells with a GD2-CAR were activated by EBV antigen and co-stimulated through the B7/CD28 axis to convert into CAR-EBV-specific T cells. Normal levels of EBV DNA instead of evident EBV-positive malignancy were sufficient for their expansion and maintenance (161). In a long-term clinical trial with 19 neuroblastoma patients, CAR-EBV-specific T cells and CAR-T cells demonstrated similar fates. Their duration of persistence was highly concordant with the proportion of helper and T central memory cells (125). Strikingly, researchers have designed a whole-cell vaccine promoting the cross-presentation of viral epitopes to the native virus-specific TCRs aiming at expanding CAR-redirected CTLs (127). They further engineered the vaccine with CD40L and OX40L to promote the overall antitumor activity of CAR-CMV-specific T cells. A phase 1 clinical trial indicated that VSTs expressing HER2-CAR were safe and beneficial for progressive glioblastoma patients (128).

### iPSCs

T cells derived from iPSCs can also be a source of CAR-T cells (162). A bank of iPSCs with common HLA haplotypes can be generated to minimize the risk of allo-rejection. CAR-T cells are generated from one clonal engineered pluripotent cell line and are therefore homogeneous. Although iPSC-T cells expressed their rearranged endogenous αβ-TCR on the surface, they acquired an innate-like phenotype instead of that of natural naive or memory CD8αβ^+^ T lymphocytes (163). Surprisingly, iPSC-derived, CAR-expressing T cells displayed an mRNA expression profile similar to that of γδ T cells (162). Under the stimulation of CD19, expanding CAR-iPSC-T cells expressed NK receptors such as NKp44, NKp46, and NKG2D and switched to the Th1 phenotype. The rate of CAR expression on iPSC-T cells was relatively lower than that on γδ or αβ T cells, which might reduce tonic signaling. A phase 1 study of iPSC-derived CAR-T cells targeting CD19 has been conducted for leukemia patients (164).

### CD26^high^ T Cells

It is suggested that some subsets of T cells like CD26^high^ T cells may be more efficacious than others (165). CD26 is an enzymatically active, multifunctional protein beneficial for T-cell co-stimulation as well as the binding of extracellular molecules (166). CD26 might augment antitumor immunity in an MHC-independent manner (167). CD26^high^ CD4^+^ T cells generated excessive IL-17 showing a phenotype resembling Th17, which exhibited stem cell-like qualities (168). CD26^high^ CAR-T cells with enhanced stemness and apoptosis resistance produced increased cytokines (IL-17A, IFN-γ, IL-2, TNF, and IL-22) and elevated CCR2 and CCR5 (129). Intriguingly, CD26^high^ T cells also expressed multiple co-stimulatory and co-inhibitory markers (PD-1, CD40L, OX40, and GITR). GITR is a co-stimulatory receptor enhancing effector T-cell function and alleviating T regulatory cell (Treg)-mediated immunosuppression (169), suggesting its potential to provide a co-stimulatory signal for CAR.

## Conclusion

To overcome the challenges of CAR-T cells in solid tumors and promote their commercial application, we presented the achievements of receptors, signaling, and immune cells beneficial for CAR construction. Either of the stimulation domains such as CD28/4-1BB/CD3ζ or CD4^+^/CD8^+^ T cells can be replaced for better efficacy. We reanalyzed the construct of CARs from 1G to 4G and focused on unconventional designs including specific receptors or novel co-stimulatory domains. Except for scFvs, different receptors with high affinities have emerged. Artificial switch receptors can reverse inhibitory signaling and SynNotch receptors can reduce “on-target/off-tumor” effects. Furthermore, incorporating natural TCR and NKG2D into CAR can expand the range of targets. Developments of stimulation domains have been investigated as well. Intriguingly, changing the number or location of ITAMs can promote an activation signal. In addition to CD28 and 4-1BB, more molecules, such as ICOS, CD27, and MyD88, are participating in the CAR machinery and their interaction might achieve synergistic effects. We further compared the characteristics of diverse immune cells including NK cells, NKT cells, macrophages, and several subtypes of T cells and evaluated their antitumor potential. Using virus-specific T cells or iPSC as drivers of CAR can decrease the risk of GvHD. Integrating CAR into NK cells or CIKs with NKG2D can avert off-target effects. In a word, a growing number of candidates of the optimal CAR construct without the conventional framework are appearing. Their effects need to be objectively compared and evaluated to inspire researchers.

The application of NK cells and associated receptors or co-stimulatory molecules in CAR is of great interest. As a pivotal component of the innate immune system, NK cells can show potent antitumor function without severe side effects. Their receptor NKG2D is also expressed on the surface of CIK, γδ T cells, and NKT cells. CAR that contained NKG2D can exhibit superior recognition capacity. Meanwhile, DAP12 as the adaptor of NKG2D shows vigorous activation ability. The ingenious combination of NKG2D and DAP12 or CD3ζ can maximumly activate immune cells and minimize immune suppression. For instance, CD3ζ modification brings CAR-NK cell resistance to TGF-β, which inhibits DAP10 transcription to suppress endogenous NK cells (35). Moreover, strategies of 4G CAR can be applied to CAR-NK cells. Adding IL-15 or its receptor probably extends the persistence of CAR-NK/NKT cells.

It is noteworthy that the design of CAR is undergoing changes from a single chain to double chains or from a series model to a parallel model. The recognition domain and activation domain may be separated into two chains, like KIR/TREM and DAP12, to improve responsiveness and stability. Another strategy is replacing the single chain with only one CD3ζ by two chains similar to TCR, such as STAR, AbTCR, and TRuC, which have advantages in fully stimulating immune cells. These findings suggest that using two chains with different or synergistic functions can improve CAR-T efficacy and avoid tonic signaling. Furthermore, there is still a wide research field awaiting investigation. For example, even Treg cells can express CAR and different co-stimulators might influence their functions (170). Various co-stimulatory molecules like GITR and dectin-1 deserve attention as well (171, 172).

In conclusion, assembling distinct receptors, stimulation domains, and immune cells might realize unexpected achievements. To acquire optimal CAR, multiple novel designs and collocations need to be developed.

## Author Contributions

MS planned the manuscript. TY and S-KY wrote the draft and designed the figures. YX corrected the grammar errors and notation mistakes. K-HL reviewed and revised the manuscript. All authors contributed to the article and approved the submitted version.

## Funding

This study was supported by the National Natural Science Foundation of China (82172708).

## Conflict of Interest

The authors declare that the research was conducted in the absence of any commercial or financial relationships that could be construed as a potential conflict of interest.

## Publisher’s Note

All claims expressed in this article are solely those of the authors and do not necessarily represent those of their affiliated organizations, or those of the publisher, the editors and the reviewers. Any product that may be evaluated in this article, or claim that may be made by its manufacturer, is not guaranteed or endorsed by the publisher.
